# Lignocellulose hydrolytic enzymes production by *Aspergillus flavus* KUB2 using submerged fermentation of sugarcane bagasse waste

**DOI:** 10.1080/21501203.2020.1806938

**Published:** 2020-08-18

**Authors:** Nattida Namnuch, Anon Thammasittirong, Sutticha Na-Ranong Thammasittirong

**Affiliations:** a Department of Microbiology, Faculty of Liberal Arts and Science, Kasetsart University, Nakhon Pathom, Thailand; b Microbial Biotechnology Unit, Faculty of Liberal Arts and Science, Kasetsart University, Nakhon Pathom, Thailand

**Keywords:** Cellulase, xylanase, *Aspergillus flavus*, lignocellulose, sugarcane bagasse

## Abstract

Lignocellulosic wastes, rice straw, sugarcane bagasse, rice bran and sawdust, and pure commercial carboxymethyl cellulose (CMC) and xylan were used as substrates to cultivate cellulolytic fungus, *Aspergillus flavus* KUB2, in submerged fermentation at 30°C. Of all the substrates, sugarcane bagasse was a good source for the production of cellulolytic and also hemicellulolytic enzymes. The maximum activities of endoglucanase (CMCase), total cellulase (FPase) and xylanase using sugarcane bagasse as substrate were 8%, 75% and 165%, respectively, higher than those of the commercial substrates. The time course determination of enzyme production revealed that the highest CMCase (1.27 U/ml), FPase (0.72 U/ml) and xylanase (376.81 U/ml) activities were observed at 14 days of fermentation. Fourier transform infrared (FTIR) spectroscopy and scanning electron microscopy (SEM) analyses confirmed the efficient structural alteration of sugarcane bagasse caused by enzymatic actions during *A. flavus* KUB2 cultivation. Based on the results of the hydrolytic enzyme activities, FTIR and SEM, *A. flavus* KUB2 is suggested as an efficient hydrolytic enzymes producer and an effective lignocellulose degrader, while sugarcane bagasse can be applied as a low-cost carbon source for the economical production of lignocellulose hydrolytic enzymes by *A. flavus* KUB2.

## 1. Introduction

Lignocellulosic biomass is considered a promising sustainable resource for the production of pharmaceuticals and chemicals to replace fossil resources. Lignocellulose is a complex structure composed of lignin (25–30%), hemicellulose (25–30%) and cellulose (35–50%) (Hagen 2015). Cellulose, the major structural component of the higher plant cell wall, represents the most abundant organic compound available on earth and is the principal waste from agricultural residues as well as a renewable source of energy. Cellulose is a polysaccharide composed of a linear chain of β-1,4-glycosidic linked D-glucose units. Cellulase is an enzyme complex system, which consists of exoglucanase, endoglucanase and β-glucosidase, that hydrolyses the β-1,4 linkages of the cellulose chain into glucose (Kuhad et al. 2016). Cellulases are widely used such as in the food, fuel, textiles, pulp and paper industries (Bhat 2000; Bano et al. 2019).

A wide variety of microorganisms including fungi and bacteria have been recognised for their ability to hydrolyse lignocellulosic materials. In particular, filamentous fungi have had a crucial role in the majority of natural biomass degradation. The *Aspergillus* genus is one of the major microbial agents of decay and decomposition and is the most common source of the production of cellulolytic enzymes (Sarkar and Aikat 2014; Kumar et al. 2016; Sohail et al. 2016). Cellulase production has been reported for several *Aspergillus* species. *A. fumigatus* CWSF-7 isolated from soil contaminated with lignocellulosic waste was observed to produce high titre of cellulases. The maximum endoglucanase (CMCase) activity and total cellulase (FPase) activity obtained were 1.9 U/ml and 0.9 U/ml, respectively (Mohapatra et al. 2018). *A. terreus* MS105 had the highest CMCase (0.47 U/ml) and FPase (0.29 U/ml) activities when cultured in medium containing CMC (Sohail et al. 2016). *A. flavus* AT-2 produced CMCase (5.1 IU/ml) and FPase (0.56 IU/ml) under submerged fermentation using rice straw as substrate (Dutt and Kumar 2014). Besides the ability to produce cellulolytic enzymes, *Aspergillus* species have also been reported to produce hemicellulolytic enzymes. de Alencar Guimaraes et al. (2013) reported that *A. niger* and *A. flavus* produced xylanase using various agro-industrial and commercial substrates. Ferreira et al. (2018) found that *A. japonicas* Saito produced the highest xylanase activity (3.55 U/ml) when sugarcane bagasse was used as substrate. *A. niger* A12 produced maximum CMCase and xylanase activities of 432 U/l (0.432 U/ml) and 714 U/l (0.714 U/ml), respectively, under submerged cultivation with sugarcane bagasse as substrate (Cunha et al. 2012).

The present study isolated fungi from soil and decaying wood and screened them for cellulolytic activity on CMC agar and CMC broth. The selected cellulolytic fungal isolate, *Aspergillus flavus* KUB2, was culture in defined substrates and lignocellulosic wastes and evaluated crude enzyme for cellulolytic and also hemicellulolytic activities. After *A. flavus* KUB2 cultivation, lignocellulosic substrates were characterised using Fourier transform infrared (FTIR) spectroscopy and scanning electron microscopy (SEM) and compared with substrate without fungal inoculation.

## 2. Materials and methods

### 2.1. Fungal isolation

Fungal isolates were isolated from soil-containing decomposed plant materials and wood decayed samples. A 10 g of each sample was added to 90 ml of sterile deionised water and mixed for 5 min. After serial dilutions, the suspensions were spread on potato dextrose agar (PDA) supplemented with 100 µg/ml streptomycin and incubated at 30°C for 5–7 days. Further separation was carried out until pure cultures were obtained.

### 2.2. Qualitative and quantitative screening of cellulolytic fungi

All fungal isolates were cultured on carboxymethyl cellulose (CMC) agar. The medium was prepared as described by Irfan et al. (2017) with slight modification of the composition (5.0 g/l CMC, 1.0 g/l (NH_4_)_2_SO_4_, 1.0 g/l KH_2_PO_4_, 1.0 g/l KCl, 0.5 g/l MgSO_4_•7H_2_O, 0.5 g/l yeast extract, and 15.0 g/l agar). The pH was adjusted to 5 prior to sterilisation. Inoculation was carried out by placing a 5 mm plug of 5-day-old culture on the centre of the CMC agar medium and incubating at 30°C for 4 days. The growth of fungi was measured based on the diameter of the colony. A 10 ml volume of 0.5% Congo red was then added to each plate. After 15 min, the solution was discarded and the cultures were washed with 10 ml of 1 M NaCl. The observed clear hydrolytic zone around the fungal colony indicated cellulase production. The fungal isolates producing a clear hydrolytic zone were selected for further quantitative screening in liquid medium.

The quantitative screening was carried out using submerged cultivations in Erlenmeyer flasks containing 50 ml of basal medium (1.4 g/l (NH_4_)_2_SO_4_, 2.0 g/l KH_2_PO_4_, 0.4 g/l CaCl_2_•2H_2_O, 0.3 g/l MgSO_4_•7H_2_O, 1.56 mg/l MnSO_4_•H_2_O, 5.0 mg/l FeSO_4_•7H_2_O, 1.4 mg/l ZnSO_4_•7H_2_O and 2.0 mg/l CoCl_2_) (Ja’afaru 2013) containing 10.0 g/l of CMC (pH 5.0). After sterilisation of the basal medium, the flasks were incubated with 5 plugs (0.5 mm diameter) of the fungal mycelia grown on PDA medium and incubated at 30°C with shaking at 150 rpm. The crude enzyme was collected at 7 days of incubation using centrifugation at 10,000 rpm and 4°C for 15 min and analysed for CMCase activity.

### 2.3. Fungal identification and phylogenetic analysis

Total DNA was extracted following the method described by Gonzalez-Mendoza et al. (2010). Amplification of the internal transcribed spacer (ITS) region was performed using PCR with the universal primers ITS1 and ITS4, according to White et al. (1990). PCR was performed in 25 μl reaction tube containing, 0.2 µM of each primer, 1 U of *Taq* DNA polymerase, 0.2 mM of each dNTP, 2.5 mM of MgCl2, 1× PCR reaction buffer and approximately 10 ng of DNA template. Thermal cycling condition was as follows: denaturation at 95°C for 8 min followed by 30 cycles of denaturation at 95°C for 1 min, annealing at 57°C for 1 min and extension at 72°C for 1 min and incubated at 72°C for 8 min.

To identify *Aspergillus* spp. amplification of a partial β-tubulin (*BenA*) and calmodulin (*CaM*) genes was performed using primer pair Bt2a and Bt2b (Glass and Donaldson 1995) and cmd5 and cmd6 (Hong et al. 2005), respectively. The PCR reaction solution was performed as described above. Thermal cycling condition was as follows: denaturation at 94°C for 5 min followed by 30 cycles of denaturation at 94°C for 45 s, annealing at 55°C for 45 s and extension at 72°C for 1 min and incubated at 72°C for 7 min. The DNA sequences were analysed with other related sequences published in GenBank using the BLASTN programme. The ITS, *BenA* and *CaM* sequences were deposited in the GenBank database under accession numbers: MN880871, MT774497 and MT774250, respectively. The sequences were aligned using the ClustalW software and phylogenetic trees were conducted using the MEGA X programme (Kumar et al. 2018).

### 2.4. Primary screening for cellulase activity at different levels of pH and temperature

The fungus, *A. flavus* KUB2, was cultured as described above on CMC agar plates at pH levels of 4.5, 5.0, 5.5, 6.0, 6.5, 7.0, 7.5 or 8.0 and incubated at temperatures of 25°C, 28°C or 30°C for 4 days. After staining with Congo red, the clear hydrolytic zone around the fungal colony was measured for calculation of the enzymatic index (EI) using the following formula (Florencio et al. 2012):

EI = diameter of hydrolysis zone/diameter of colony

### 2.5. Hydrolytic enzymes production using submerged fermentation

The 5 plugs of *A. flavus* KUB2 were inoculated into the fermentation medium (1 g/l KH_2_PO_4_, 0.5 g/l MgSO_4_•7H_2_O, 5 g/l peptone and 5 g/l yeast extract) (Kumar et al. 2016) supplemented with 20 g/l of each lignocellulosic waste, rice straw, sugarcane bagasse, rice bran and sawdust, or 10 g/l of either of the commercial substrates (CMC and xylan) at pH 7.0. After culture at 30°C with shaking at 150 rpm for 7 days, the crude enzyme was collected using centrifugation at 10,000 rpm for 15 min at 4°C and analysed for CMCase, FPase and xylanase activities.

The time course of enzyme production was performed using fermentation medium supplemented with 20 g/l sugarcane bagasse. *A. flavus* KUB2 was cultured as described above, samples were withdrawn at 5, 7, 10, 14 and 21 days of incubation and centrifuged at 10,000 rpm for 15 min at 4°C to obtain crude enzyme for analysis of the CMCase, FPase and xylanase activities.

### 2.6. Enzyme assay

CMCase was determined following the method of Ghose (1987) with slight modification. The assay mixture consisted of 0.5 ml of diluted crude enzyme and 0.5 ml of 1% CMC in 0.05 M sodium citrate buffer at pH 4.8 and was incubated at 50°C for 30 min. The FPase activity was determined according to the method of NREL (Adney and Baker 2008). The reaction mixture contained 0.5 ml of diluted crude enzyme and a Whatman filter paper No. 1 strip (1*6 cm) in 1 ml of 0.05 M sodium citrate buffer at pH 4.8 and was incubated at 50°C for 60 min. All enzyme reactions were stopped by the addition of 3 ml of 3,5-dinitrosalicylic acid and then were immediately boiled for 5 min. The released reducing sugars were measured spectrophotometrically at 540 nm. Glucose standard curve was used to calculate the CMCase and FPase activities. One unit (U) of enzyme activity was defined as the amount of enzyme that released 1 µmole of reducing sugar equivalence as glucose per min. The xylanase activity was determined as described by Bailey et al. (1992). The reaction mixture contained 0.2 ml of crude enzyme and 1.8 ml of substrate in 0.05 M sodium citrate buffer at pH 5.3 and was incubated at 50°C for 5 min. The enzyme reactions were stopped by the addition of 3 ml of 3,5-dinitrosalicylic acid and then were immediately boiled for 5 min. The released reducing sugars were measured spectrophotometrically at 540 nm. Xylose standard curve was used to calculate the xylanase activity. One unit (U) of enzyme activity was defined as the amount of enzyme that released 1 µmole of reducing sugar equivalence as xylose per min.

### 2.7. Fourier transform infrared (FTIR) spectroscopy

The chemical structures of sugarcane bagasse were determined using FTIR (Vortex 70, Bruker, Germany). The spectra were measured at a spectral solution of 4 cm^−1^ with an average of 60 scans from 2000 to 400 cm^−1^.

### 2.8. Scanning electron microscopy (SEM) analysis

The surface morphology of sugarcane bagasse was observed using a scanning electron microscope (Apreo, FEI, Czech Republic) with an operating voltage of 5 kV.

## 3. Results and discussion

### 3.1 Screening of cellulolytic fungi and identification

**Figure 1.**
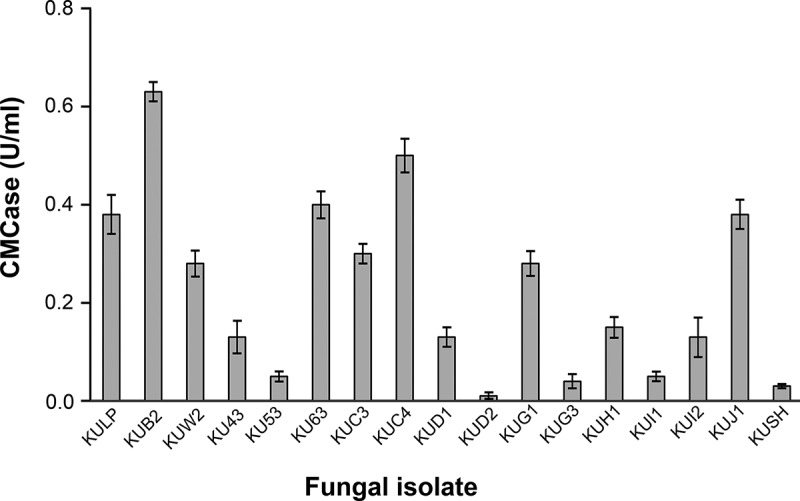
CMCase activity of fungal isolates from quantitative screening under submerged fermentation using CMC as substrate

In total, 57 fungal isolates were isolated from the soil containing decomposed plant materials and decayed wood samples. Initial screening of the cellulase-producing fungi was carried out on agar plates using CMC as the sole carbon source and cellulase activity was detected using staining with Congo red. This qualitative determination was based on the interaction of Congo red with intact β-D-glucan in CMC, while the area with the degradation of cellulose by the enzymes resulting in clear zones or the appearance of a pale halo (Lamb and Loy 2005; Florencio et al. 2012). Of all fungal isolates, 17 isolates displayed cellulolytic activity based on the formation of clear zones on the CMC agar and were further confirmed for CMCase production in basal liquid medium containing 1% CMC as the sole carbon source. As shown in Figure 1, it was clear that all fungi had CMCase activities but at various levels. The fungal isolate KUB2, isolated from soil, had the highest CMCase activity (0.63 U/ml).

**Figure 2.**
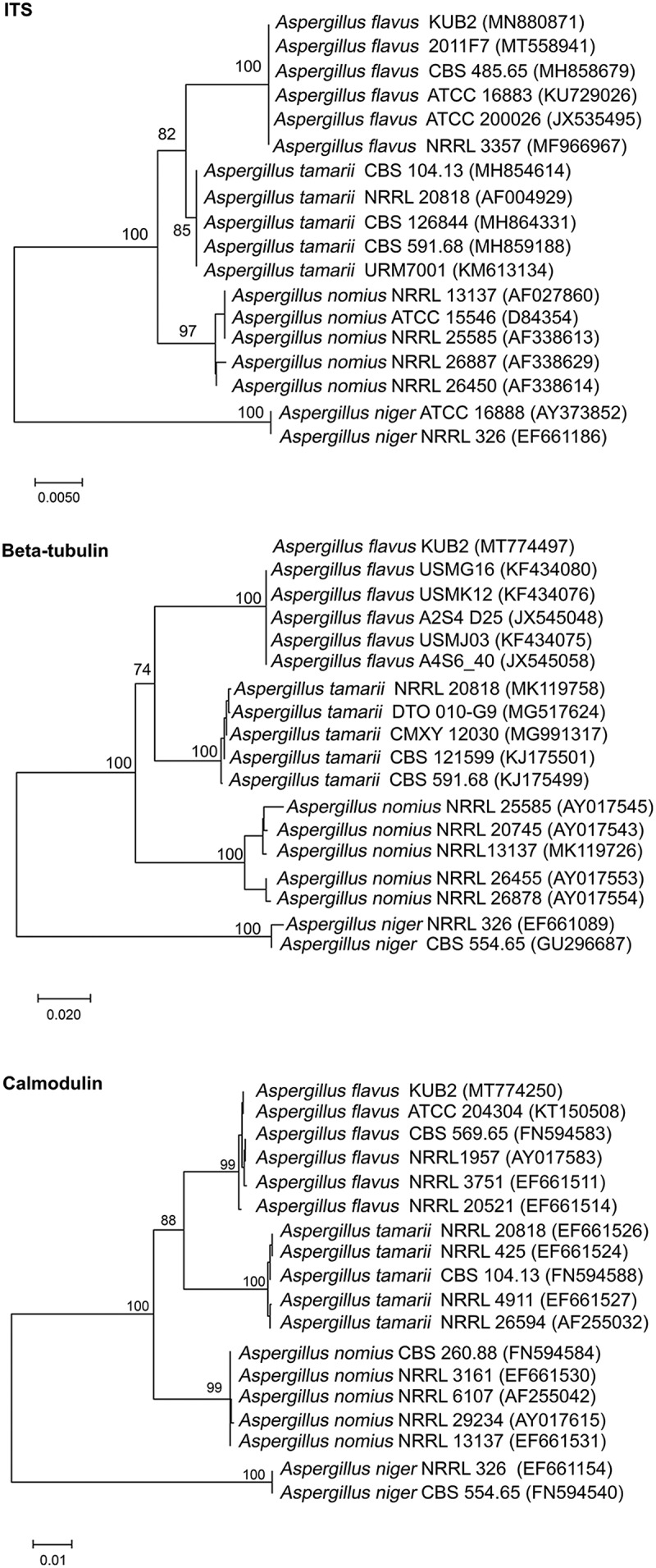
Phylogenetic trees based on ITS, *β*-tubulin and calmodulin gene sequences of *A. flavus* KUB2

This fungus was identified at the molecular level as *Aspergillus flavus* KUB2 and phylogenetic tree analysis based on ITS, β-tubulin and calmodulin gene sequences indicated that this fungus was most closely related to *A. flavus* (Figure 2). The ITS, *BenA* and *CaM* sequences were deposited in the GenBank database under accession numbers: MN880871, MT774497 and MT774250, respectively.

### 3.2. Effect of pH and temperature on growth and cellulolytic activity of A. flavus KUB2

Plate screening assay based on the hydrolysis activity of the enzymes in the CMC medium was used to initially study the effect of pH and temperature on the growth and cellulolytic activity of *A. flavus* KUB2. In this study, *A. flavus* KUB2 was cultured on CMC agar at different pH levels (4.5–8.0) and incubated under different temperature conditions (25°C, 28°C and 30°C). Measurements of growth colonies and hydrolysis zones were used to evaluate the cellulase activity using the enzymatic index (EI) which is a semi-quantitative, rapid, simple and practical method for selecting and comparing the potential of microbial enzyme production. Microbial isolates with high EI values were considered to have potential for hydrolytic enzyme production (Florencio et al. 2012; Bedade et al. 2017; Saroj and Narasimhulu 2018). The results revealed that *A. flavus* KUB2 could grow on CMC agar at all temperatures and pH levels. However, *A. flavus* KUB2 produced a clear cellulolytic zone only with cultivation at 30°C, which produced maximum EI values with pH levels of 7 (1.12), 7.5 (1.11) and 8 (1.10) as shown in Table 1. These results demonstrated that the cultivation pH and temperature (especially the temperature) are critical for cellulase activity by *A. flavus* KUB2. No cellulase activity by *A. flavus* KUB2 was detected at an incubation temperature lower than 30°C perhaps because of lower transportation of substrate across the cells at lower temperatures, resulting in a lower yield of the products (Dutt and Kumar 2014).

**Table 1.** Enzymatic indices of *A.* *flavus* KUB2 on CMC agar, under different pH and temperature conditions, stained with Congo red

Growth condition	Colony diameter (cm)	Hydrolysis zone diameter (cm)	Enzymatic index (EI)
25°C			
pH 4.5	3.65 ± 0.07^ab^	-	-
pH 5.0	3.75 ± 0.07^a^	-	-
pH 5.5	3.83 ± 0.04^a^	-	-
pH 6.0	3.60 ± 0.21^abc^	-	-
pH 6.5	3.58 ± 0.11^abc^	-	-
pH 7.0	3.43 ± 0.04^bc^	-	-
pH 7.5	3.36 ± 0.13 ^c^	-	-
pH 8.0	3.15 ± 0.07^d^	-	-
28°C			
pH 4.5	4.11 ± 0.13^b^	-	-
pH 5.0	4.20 ± 0.14^ab^	-	-
pH 5.5	4.31 ± 0.01^a^	-	-
pH 6.0	4.20 ± 0.07^ab^	-	-
pH 6.5	4.18 ± 0.04^ab^	-	-
pH 7.0	3.92 ± 0.00 ^c^	-	-
pH 7.5	3.93 ± 0.04 ^c^	-	-
pH 8.0	3.85 ± 0.00 ^c^	-	-
30°C			
pH 4.5	4.60 ± 0.07^a^	4.80 ± 0.07^a^	1.04 ± 0.00^d^
pH 5.0	4.19 ± 0.02^b^	4.39 ± 0.05 ^c^	1.05 ± 0.02 ^cd^
pH 5.5	4.23 ± 0.11^b^	4.60 ± 0.07^b^	1.09 ± 0.01^ab^
pH 6.0	4.28 ± 0.04^b^	4.50 ± 0.00^b^	1.05 ± 0.01^bcd^
pH 6.5	4.15 ± 0.00^b^	4.50 ± 0.07^b^	1.08 ± 0.02^abc^
pH 7.0	3.90 ± 0.14 ^c^	4.38 ± 0.11 ^c^	1.12 ± 0.01^a^
pH 7.5	3.73 ± 0.04 ^c^	4.15 ± 0.00^d^	1.11 ± 0.01^a^
pH 8.0	3.78 ± 0.18 ^c^	4.15 ± 0.07^d^	1.10 ± 0.03^a^

Data represent the mean±standard deviation. At each temperature, different lowercase superscript letters in each column indicate significant differences among the pH levels (*p* < 0.05).

### 3.3. Hydrolytic enzymes production of the fungal isolate

The use of low-cost substrates for cultivating microorganism provide economical enzyme production. In this study, lignocellulosic wastes, rice straw, sugarcane bagasse, rice bran and sawdust, and pure commercial CMC and xylan were used as substrates to cultivate *A. flavus* KUB2 using submerged fermentation at 30°C. Several studies have reported the capability of *Aspergillus* spp. to produce cellulase and xylanase enzymes simultaneously (Ang et al. 2013; Lin et al. 2017; Ferreira et al. 2018). Therefore, the crude enzyme produced from *A. flavus* KUB2 cultivated at 7 days was evaluated for both cellulolytic enzymes (CMCase and FPase) and a hemicellulolytic enzyme (xylanase).

As shown in Table 2, the highest cellulase activities (CMCase 1.04 U/ml and FPase 0.21 U/ml) were detected in the medium composed of sugarcane bagasse followed by pure CMC, which had a maximum CMCase activity of 0.96 U/ml and FPase with maximum activity of 0.12 U/ml. The most significant xylanase activity was also observed on medium containing sugarcane bagasse (258.38 U/ml) followed by rice straw (118.56 U/ml) and pure xylan (97.53 U/ml). Sawdust was the worst at stimulating activity of CMCase (0.06 U/ml), FPase (0.01 U/ml) and xylanase (14.07 U/ml) by *A. flavus* KUB2. Numerous studies on the production of cellulase and xylanase using various carbon sources and microorganisms have reported different yields. *A. fumigatus* N2, isolated from decaying wood, had the highest CMCase activity (5.6 U/ml) and xylanase activity (91.9 U/ml) when barley straw was used as substrate (Lin et al. 2017). de Alencar Guimaraes et al. (2013) reported that the xylanase activity of *A. flavus* was detected in medium containing pure xylan from oat spelt (11.7 U/ml), while sugarcane bagasse had xylanase activity of 5.53 U/ml. *A. flavus* MTCC 9390 when cultivated using pure birch wood xylan and oat bran as substrates had xylanase activity levels of 27.28 U/ml and 25.54 U/ml, respectively (Bhushan et al. 2012). *Thermoascus aurantiacus* had CMCase activity of 60 U/ml and xylanase activity of 107 U/ml based on corncob as a substrate, but eucalyptus sawdust resulted in CMCase and xylanase activities of 0.3 U/ml and 37 U/ml, respectively (Silva et al. 2005). Besides the microorganism and cultivation condition, hydrolytic enzyme production is influenced by substrate properties such as the physical associations between the structural components, chemical composition and presence of some nutrients (Dutt and Kumar 2014; Kogo et al. 2017).

**Table 2.** Cellulase and xylanase activity of crude enzyme from *A.* *flavus* KUB2 obtained using submerged fermentation with different substrates after 7 days of fermentation

Substrate	Enzyme	Activity (U/ml)
CMC	CMCase	0.96 ± 0.06^b^
	FPase	0.12 ± 0.01^B^
	Xylanase	21.56 ± 0.85*^e^*
Xylan	CMCase	0.03 ± 0.01^e^
	FPase	0.03 ± 0.01^D^
	Xylanase	97.53 ± 2.90 *^c^*
Rice straw	CMCase	0.18 ± 0.00^d^
	FPase	0.08 ± 0.00 ^C^
	Xylanase	118.56 ± 0.85*^b^*
Sugarcane bagasse	CMCase	1.04 ± 0.02^a^
	FPase	0.21 ± 0.05^A^
	Xylanase	258.38 ± 17.36*^a^*
Saw dust	CMCase	0.06 ± 0.03^e^
	FPase	0.01 ± 0.00^E^
	Xylanase	14.07 ± 2.96 *^f^*
Rice bran	CMCase	0.47 ± 0.01 ^c^
	FPase	0.09 ± 0.01 ^C^
	Xylanase	25.75 ± 0.85*^d^*

Data represent the mean±standard deviation. Different lowercase superscript letters in a column indicate significant differences of CMCase activity among substrates (*p* < 0.05). Different uppercase superscript letters in a column indicate significant differences of FPase activity among substrates (*p* < 0.05). Different lowercase italic superscript letters in a column indicate significant differences of xylanase activity among substrates (*p* < 0.05).

**Figure 3.**
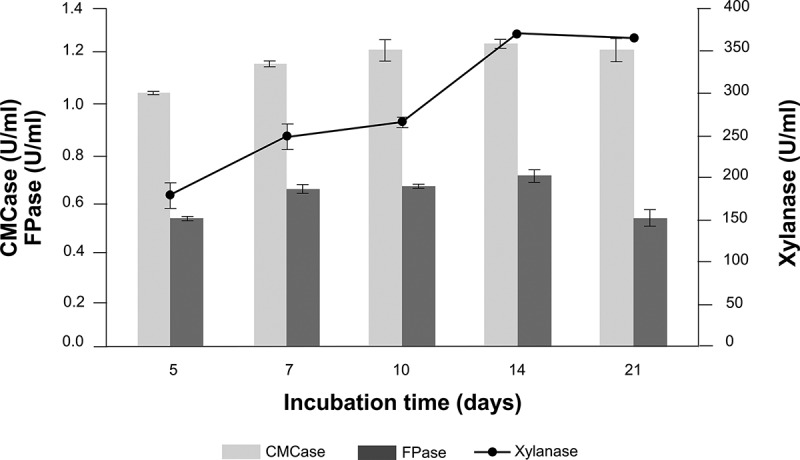
Time course of cellulases and xylanase activities from *A. flavus* KUB2 under submerged fermentation using sugarcane bagasse as substrate

With regard to the enzyme activities in the current study, sugarcane bagasse was recorded as the best source for cellulolytic and hemicellulolytic enzymes production compared with other lignocellulosic wastes and pure commercial substrates. Therefore, sugarcane bagasse was selected as the sole carbon source for further determination on the time course of hydrolytic enzyme production in submerged fermentation by *A. flavus* KUB2. Maximum activities of CMCase (1.27 U/ml), FPase (0.72 U/ml) and xylanase (376.81 U/ml) were observed at 14 days of incubation (Figure 3). Any further increase in the fermentation time caused decreased enzyme activity. The decline in enzyme production may have been due to protease production in the medium and the depletion of nutrients (Sharma et al. 2017; Bedade et al. 2017).

### 3.4. Structural change of sugarcane bagasse after *A. flavus* KUB2 cultivation

**Figure 4.**
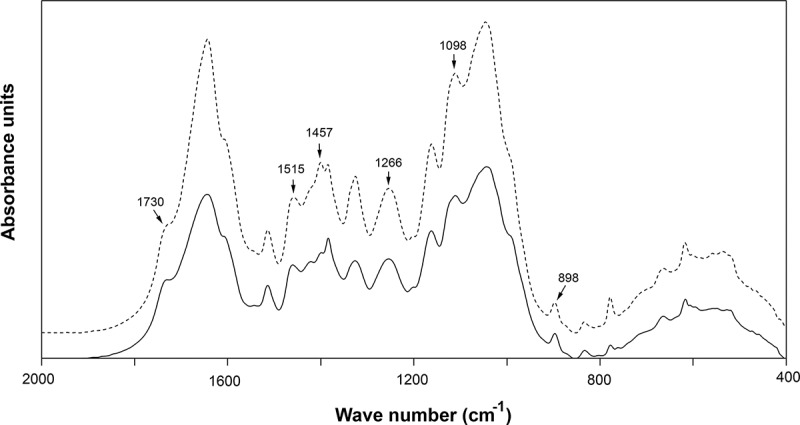
FTIR spectra of sugarcane bagasse without *A. flavus* KUB2 inoculation (dashed line) and after *A. flavus* KUB2 cultivation (solid line)

FTIR spectroscopy and SEM analyses were used to examine the main structural transformations caused by the *A. flavus* KUB2 enzymatic actions in sugarcane bagasse after 21 days of fungal cultivation in submerged fermentation. FTIR, a simple and rapid technique, is well known for qualitative and quantitative determination of lignocellulosic components. Characteristic peaks associated with cellulose, hemicellulose and  lignin could be observed at 1730 cm^-1^ (unconjugated C=O in xylan), 1515 cm^-1^ (aromatic skeleton vibrations in lignin), 1457 cm^-1^ (CH_2_ deformation stretching in lignin and xylan), 1266 cm^-1^ (syringyl ring breathing and C-O stretching in lignin and xylan), 1098 cm^-1^ (crystalline cellulose), and 898 cm^-1^ (β-glycosidic linkage) (Herrera-Franco and Valadez-Gonzalez 2005; Shi and Li 2012; Castoldi et al. 2014; Corrêa et al. 2016; Xu et al. 2017).Table 3 shows the results of the analysis of the spectra corresponding to cellulose, hemicellulose and lignin, in terms of the percentage modifications of substrate after fungal cultivation relative to the substrate without fungal inoculation and Figure 4 shows the FTIR spectral analysis. The diminutions of the peak intensities at 1098 cm^−1^ and 898 cm^−1^ indicated cellulose degradation. The decreases in the peak intensities at 1730 cm^−1^, 1457 cm^−1^ and 1266 cm^−1^ indicated hemicellulose degradation. The decreases in the peak intensities at 1515 cm^−1^, 1457 cm^−1^ and 1266 cm^−1^ suggested substantial degradation of lignin. Similar FTIR results have been reported by Xu et al. (2017) for wheat straw, rice straw and corn stover degradations by *Inonotus obliguus* under submerged fermentation and by Corrêa et al. (2016) for corncob after *Pleurotus pulmonarius* cultivation.

**Table 3.** Diminution in specific peaks in FTIR spectra of sugarcane bagasse caused by *A.* *flavus* KUB2 cultivation

Wave number (cm^−1^)	Functional groups	Diminution (%)
1730	Unconjugated C = O in xylan	27.27 ± 1.20
1515	Aromatic skeleton vibrations in lignin	30.00 ± 2.75
1457	CH_2_ deformation stretching in lignin and xylan	30.77 ± 0.85
1266	Syringyl ring breathing and C-O stretching in lignin and xylan	30.77 ± 1.95
1098	Crystalline cellulose	37.50 ± 1.00
898	β-glycosidic linkage	33.00 ± 1.15

**Figure 5.**
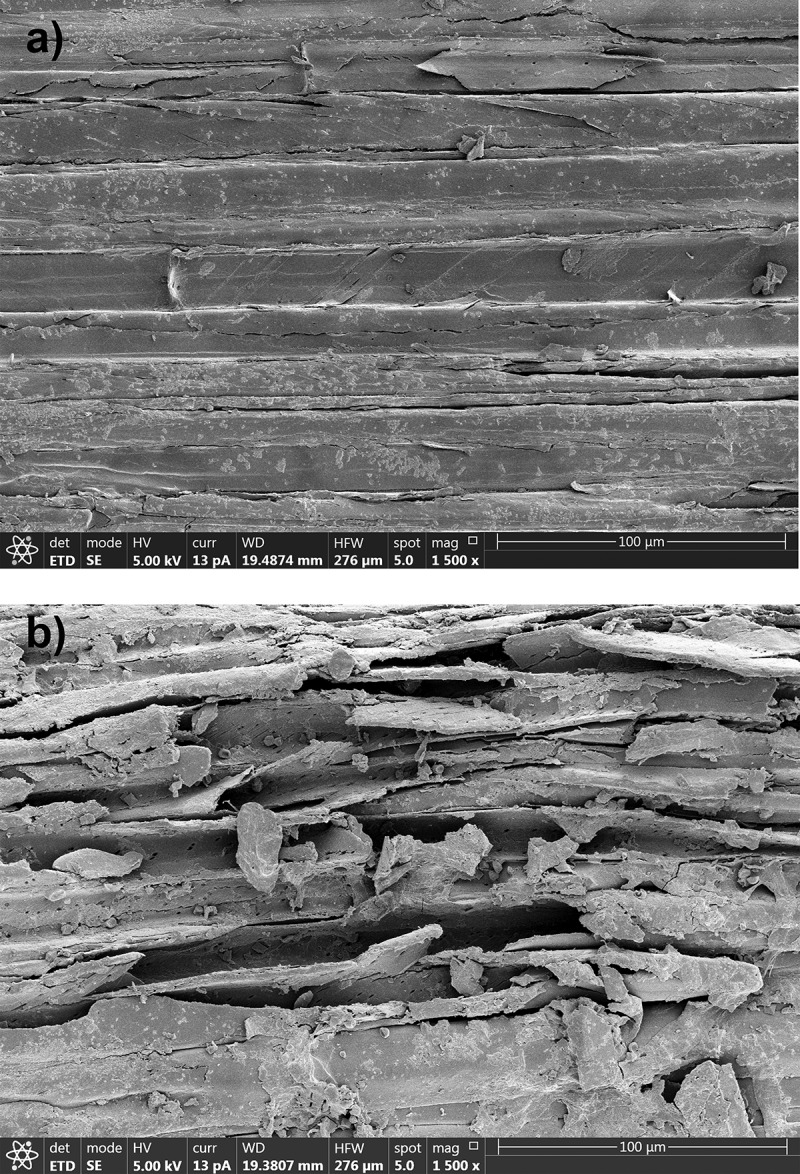
SEM of sugarcane bagasse without *A. flavus* KUB2 inoculation (a) and after *A. flavus* KUB2 cultivation (b)

Figure 5 provides the SEM images of the structural morphology of sugarcane bagasse after *A. flavus* KUB2 cultivation and of sugarcane bagasse without fungal inoculation, as the control. The sugarcane bagasse surface which was not affected by fungal cultivation had a flat and smooth surface. In contrast, the surface of sugarcane bagasse after *A. flavus* KUB2 cultivation had cracks and a large number of visible holes. Castoldi et al. (2014) reported pore formation in the wall surfaces of *Eucalyptus grandis* sawdust after cultivation with *Ganoderma lucidum, Phanerochaete chrysosporium, Pleurotus pulmonarius* and *Trametes* sp. after 30 days on solid fermentation. Xu et al. (2017) reported cracks and pores in wheat straw, rice straw and corn stover after submerged fermentation by *I. obliquus*, which resulted from a partial break down of the lignin, cellulose and hemicellulose. Nazarpour et al. (2013) reported a partially broken surface of rubberwood which resulted from the removal of lignin and the breaking of lignocellulosic networks during pretreatment with *Ceriporiopsis subvermispora*.

Numerous studies have reported that *Aspergillus* spp. are capable of producing a multi-enzymatic extracellular complex efficiently degrading lignocellulosic components in plant biomass (Ang et al. 2015; Ferreira et al. 2018). The appearance of pores detected using microscopic analysis was in agreement with a decrease in the intensities of the cellulose, hemicellulose and lignin peaks in the FTIR analysis. The FTIR and SEM results clearly demonstrated the degradation of the three major lignocellulosic components by the complex enzymes produced by *A. flavus* KUB2 during cultivation. The results of hydrolytic enzymes activities based on FTIR and SEM analyses suggested that *A. flavus* KUB2 was an efficient hydrolytic enzymes producer and an effective lignocellulose degrader of agricultural residues under submerged fermentation. Beside cellulase and xylanase studies, future research needs to study the ability of *A. flavus* KUB2 regarding ligninolytic enzymes production and to investigate the optimisation of process parameter for enhanced lignocellulolytic enzymes production.

## 4. Conclusion

In this study, *A. flavus* KUB2 isolated from soil was a potential cellulase and xylanase producer. Sugarcane bagasse was a promising carbon source for submerged fermentation to produce high activities of CMCase (1.27 U/ml), FPase (0.72 U/ml) and xylanase (376.81 U/ml) at 14 days of fermentation. Additionally, *A. flavus* KUB2 also was efficient at the degradation of lignocellulosic biomass.
